# Reviewed article: Mattos CDCL, Tomazelli J, Girianelli VR. Trends and characteristics of congenital syphilis in children under 1 year of age: a time series analysis, municipality of Rio de Janeiro, 2007-2021. Epidemiol Serv Saude. 2026:35;e20250109

**DOI:** 10.1590/S2237-96222026v35e20250109.a

**Published:** 2026-04-17

**Authors:** Cristina Pissetti

**Affiliations:** 1 Universidade Federal da Paraíba, João Pessoa, PB, Brazil Universidade Federal da Paraíba João Pessoa PB Brazil

## Peer review A

## Questionnaire

Does the manuscript contain new and significant information to justify publication? Yes

Does the Abstract (Summary) clearly and accurately describe the content of the article? Yes

Is the problem significant and concisely stated? Yes

Are the methods described comprehensively? Yes

Are the interpretations and conclusions justified by the results? Yes

Is adequate reference made to other work in the field? No

## Manuscript Structure

Length of article is: Adequate

Number of tables is: Adequate

Number of figures is: Too few

Please state any conflict(s) of interest that you have in relation to the review of this paper. None

Interest: Excellent

Quality: Good

Originality: Good

Overall: Excellent

Recommendation: Minor Revision

## Comments

O texto apresenta uma boa qualidade. A forma como os resultados foram apresentados chama a atenção para o problema estrutural da associação de cor/raça com piores indicadores (escolaridade, consultas pré-natal e incidência de sífilis). Abaixo, seguem algumas sugestões:

1. Atualizar as referências bibliográficas, priorizando artigos científicos. Neste sentido, pode haver a necessidade de adequar a discussão.

2. As figuras 2 e 3 poderiam ser omitidas e melhor descritas no texto, a fim de facilitar a compreensão para leitores não residentes no município do Rio de Janeiro.

Abaixo, algumas sugestões de artigos mais recentes:

* ANÁLISE DA SÍFILIS CONGÊNITA E SUA CORRELAÇÃO COM A ATENÇÃO À SAÚDE DA MULHER NO

MUNICÍPIO DE BOM JESUS DO ITABAPOANA - RJ.

* Spatial scenery of congenital syphilis in Brazil between 2007 and 2018: an ecological study

* Knowledge on syphilis and follow-up of pregnant in a clinic of the family of the West Zone of Rio de Janeiro

